# Gravity filtration of environmental DNA: A simple, fast, and power-free method

**DOI:** 10.1016/j.mex.2022.101838

**Published:** 2022-08-30

**Authors:** Shin-ichiro Oka, Masaki Miya, Tetsuya Sado

**Affiliations:** a Okinawa Churashima Research Center, Okinawa, Japan; b Natural History Museum and Institute, Chiba, Japan

**Keywords:** Edna, Filter cartridge, Biodiversity monitoring

## Abstract

Filtration is required during the collection of trace amounts of environmental DNA (eDNA) from water samples to achieve a concentration sufficient for downstream molecular experiments. To date, collected water samples have been filtered by humans or electric power using various instruments. We developed a simple gravity filtration system that does not need for an external force. The system comprises a plastic bag filled with a water sample (1 L), a filter cartridge, and a long plastic tube (e.g., 2 m). When hung at a height equal to the tube length, this filtration unit can enable power-free collection and concentration of eDNA at any remote location within a reasonable time (10–60 min).

• A simple, rapid, power-free, practical filtration system for environmental DNA analysis is reported.
• If there is a place to hang the filtration system, filtration can be performed anywhere.
• The filtration speed increased when the system was hung higher.

## Graphical abstract

**Figure:**
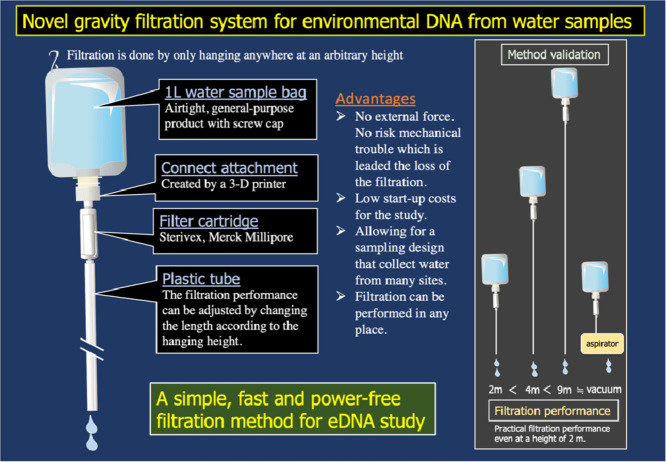
Flow diagram of experimental setup

Specifications table

**Table:** 

Subject Area:	Biochemistry, Genetics and Molecular Biology
More specific subject area:	Environmental DNA
Method name:	Gravity filtration system for environmental DNA
Name and reference of original method:	M. Miya, T. Minamoto, H. Yamanaka, S. Oka, K. Sato, S. Yamamoto, T. Sado, H. Doi, Use of a filter cartridge for filtration of water samples and extraction of environmental DNA, JoVE. (2016) No. https://doi.org/10.3791/54741.
Resource availability:	3D-printed part files are available for download as supplementary files

## Method details

Environmental DNA (eDNA), defined as residual extra-macro-organismal DNA, is used as a noninvasive and cost-effective marker for various aquatic organisms in water samples [1]. The filtration of water samples is an important process for concentrating a small amount of eDNA to an analyzable level [1]. Vacuum filtration is traditionally used in laboratories and the field [2]. In contrast, on-site filtration using syringes and filter cartridges is a more portable method [3]. While the former method can process numerous water samples, it requires sizeable equipment (e.g., aspirators) and power for suction filtration, whereas the latter requires sufficient manpower and time. Recently, portable gravity filtration has been used in some eDNA surveys [4,5]; however, details on this filtration system have not been presented, and its efficiency has not been examined. In this study, we developed a novel portable gravity filtration system for eDNA surveys, which allows users to filter a 1 L water samples within a short duration (10–60 min) in any remote location. This report explain detail, the gravity filtering system, the main purpose of which is to recover environmental DNA at this stage, but see Bessey et al. [6] for other passive filtration methods.

**Fig. 1:**
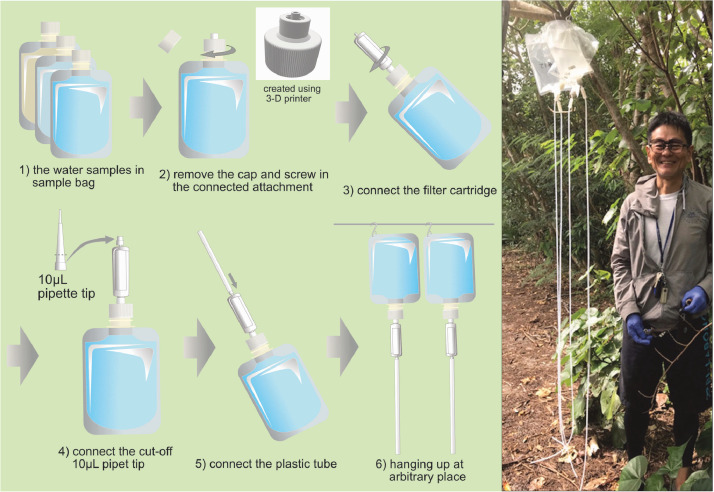
Setting protocol of the novel gravity filtration system for eDNA water samples. The photograph shows the filtration system hanging at about 2 m height in an outdoor field.

**Fig. 2:**
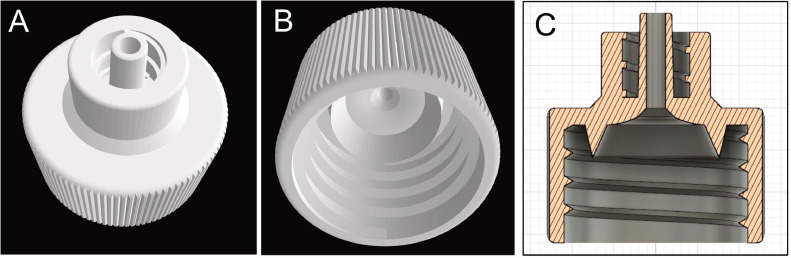
Attachment (designed with 3D CAD software) connected with a sample bag and filter cartridge. Top (A), bottom (B), and cross-section (C) views. The 3D design files (STL) are available in the supplementary files.

As shown in Fig. 1, the system consists of four parts: (1) a plastic bag (1 L, DP16-TN1000, COWPACK LTD; airtight, general-purpose product for gel or liquid foods with an attached screw cap) containing the water sample, (2) a self-made connecting attachment (Fig. 2) created with a 3D printer, (3) a Sterivex filter cartridge (pore size = 0.45 µm; Merck Millipore), and (4) a plastic tube (inner diameter: 4 mm) with an adjuster (cut-off 10 µL pipette tip base).

The printed attachment (Fig. 2) was designed to fit the plastic bag and Sterivex filter cartridge using the 3D CAD software “Fusion 360”, and were printed using the stereolithography 3D-printer “ELEGOO Mars Pro 2,” an affordable and widely available model. The printer had a production rate of 12 attachments within 3 h. The cost of resin was less than 0.5 USD per piece. The 3D design files of the attachment are available for downloading as supplementary files. This attachment is similar to the handmade cap reported in a previous study [2]; however, the 3D printer product has a more integrated structure, so there is no risk of water leakage and durability.

The filtration was performed by hanging the system at an arbitrary height (Fig. 1). Water in the bag was passed through a filter cartridge and discharged automatically through a plastic tube. The only forces applied to the filtration were the pushing and pulling forces of the water weight in the sample bag and plastic tube, respectively. Increasing the bag height was observed to increase the water weight in the plastic tube, consequently increasing the filtration speed (see the Method validation section); a height of up to approximately 2 m is manageable.

The following is a list of “notes” for this method:

• Sterilize the connecting attachment, cut-off pipette tip, and plastic tube with 10% commercial bleach (ca. 0.6% sodium hypochlorite) before use to avoid contamination.
• Tight joints are required at each connection point, or the filtration speed becomes reduced owing to the reduction in the water volume in the tube due to potential air leaks.
• A plastic tube with an inner diameter of 3 mm was attached directly to the outlet port of the Sterivex filter cartridge (outer diameter, 3.5 mm) without the cut-off pipette tip adjuster.
• A hole was made in the upper part of the hanged sample bag for air influx to prevent a decrease in filtration speed due to the negative air pressure in the bag.

The main advantages of this method are as follows:

• No external force is applied, larger pieces of equipment (such as aspirators) are not required, and filtration loss due to mechanical problems is negated.
• Low start-up costs, that is, the printed attachments can be made with an inexpensive 3D printer costing less than 300 USD with a low resin cost (less than USD 0.5/piece).
• The disposable items are minimal, and the connecting attachment and plastic tube can be reused after sterilization with bleach.
• Rapid sampling: Because there is no on-site filtration, only water sampling is required for field protocols.
• Filtration can be performed at any location, such as in a laboratory or bathroom.

This study does not cover water sampling or eDNA metabarcoding methods. These aspects should be referred to in the Environmental DNA Sampling and Experimental Manual [7].

## Method validation

**Fig. 3:**
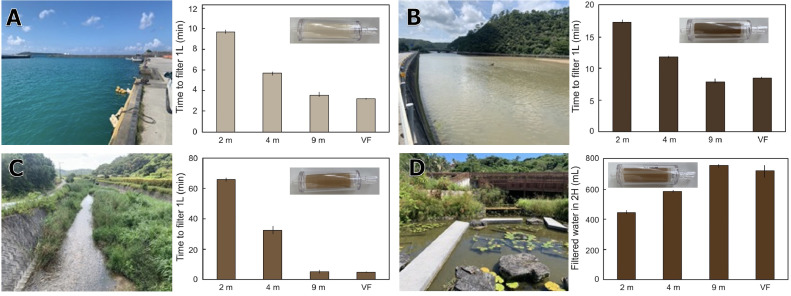
Filtration performance by gravity filtration at each height and vacuum filtration (VF). Sea (A), brackish (B), and river water (C) filtration efficiencies were estimated as the average time required to filter 1 L of sample water, and pond water (D) filtration efficiency was estimated as the average amount of water filtered in 2 h. The vertical bar indicates the range. The photographs are the filter cartridge after filtration, showing the water turbidity of each sample.

The performance of the gravity filtration system under several height conditions was compared to that of vacuum filtration using a parallel filtering system with an aspirator [2,8]. Gravity filtration was performed at heights of 2, 4, and 9 m (tube lengths downstream of the filter cartridge). The target water samples were seawater from the fishery port, brackish water from the mangrove forest, freshwater from a small river, and artificial ponds (all collected in the northern part of Okinawa Island, southern Japan). Filtration performance was measured as the time required to complete filtration of 1 L of each sample. However, because the pond water filtration lasted for more than 2 h for all methods, the amount of water filtered in 2 h was measured instead. Filtration was replicated three times under each condition. The gravity filtration performance increased as the height increased, and a height of 9 m yielded the same performance as the aspirator method. Although the filtration efficiency at 2 m, the most practical height, was the lowest, it exhibited an efficient filtration performance of 10 min and 17 min for seawater and brackish water, respectively. However, at a height of 2 m, the filtration of turbid freshwater required considerable time and hence was impractical (Fig. 3). To solve this problem, it is preferable to hang the device at a higher location or utilize vacuum filtration.

To confirm the quality of the gravity filtration samples, MiFish eDNA metabarcoding [9] was performed on actual water samples to evaluate whether sufficient fish species were detected. Three samples were gravity-filtered at a height of 2 m from 1 L of seawater obtained once a month between February and April 2022 from the coast of Tateyama, Chiba Prefecture, located along the Pacific coast of Japan. Libraries were prepared for three samples using MiFish primers [10] and subjected to MiSeq sequencing [11]. These sequences were subjected to taxon assignment [11]. We detected 27–38 fish species in the three samples, with a total of 59 species distributed across 32 families (Supplemental data; Table S1). The species richness (59 spp.) and composition of the fish communities were almost concordant with those of previous studies performed around the sampling area [11,12]. All the species detected at high frequencies were common in the region, and no species were included that were unnatural (Table S1), suggesting that the samples obtained by gravity filtration are of sufficient quality to be used for eDNA metabarcoding.

## Declaration of Competing Interest

The authors declare that they have no known competing financial interests or personal relationships that could have influenced the work reported in this study.

## Data Availability

Data will be made available on request.

## Appendix Supplementary materials

Image, application 1

Image, application 2
